# Supplementary material for: Instant classification for the spatially-coded BCI
Source: PLoS One. 2022 Apr 28;17(4):e0267548. doi: 10.1371/journal.pone.0267548 (PMC9049359; doi:10.1371/journal.pone.0267548)
Supplement: Attachment — Submitted filename: response to reviewers 2.pdf [file pone.0267548.s001.pdf]

Zentrum für Experimentelle Medizin
Institut für Neurophysiologie und Pathophysiologie

Dr. Alexander Maÿe
Wissenschaftlicher Mitarbeiter

Martinistraße 52
20246 Hamburg

Universitätsklinikum Hamburg-Eppendorf | Martinistraße 52 | 20246 Hamburg
Institut für Neurophysiologie und Pathophysiologie, N43

Gebäude Nord 43, MEG Büro 2
Raum 005
Telefon: +49 (0) 40 7410-55352
Fax: +49 (0) 40 7410-57752
a.maye@uke.de
www.uke.de

PloS ONE
Editor in Chief

Betreff: Revised manuscript PONE-D-21-13493

Hamburg, 14.2.2022

Dear Editor,

Thank you very much for reviewing our manuscript. We think that many issues raised by reviewer #2 are appropriate, and we addressed them in the revised manuscript as described below (reviewer comments in italics):

1. *The authors failed to show the novelty in their work. Please clarify the innovation in the study.*

We made the innovation more explicit in the rewritten Abstract. We think the two innovative aspects also become clear at the end of the Introduction.

2. *Please re-write the abstract as it looked a very simple one. Abstract should discuss three important things. (i). Limitations of available literature (ii) Method proposed by the author with technical information (iii) advantages and application of proposed method.*

We rewrote the abstract according to the suggested structure.

3. *I recommend authors to use multiscale principal component analysis (MSPCA) which is a combination of PCA and wavelets and useful for noise removal from network packets? The details of MSPCA can be found in "Motor imagery BCI classification based on novel two-dimensional modelling in empirical wavelet transform"*

We have received some more suggestions for alternative data processing and analysis methods during the revision process of our previous publications on the spatially-coded SSVEP BCI. We explored them all, but none of them outperformed the approach we describe in the manuscript. In addition, we also investigated new analysis methods for SSVEP BCIs like task-related component analysis (TRCA, Tanaka et al., 2013), individual-template CCA (Bin et al., 2011) or convolutional neural networks (Waytowich et al., 2018). For noise removal in SSVEP BCIs, the maximum signal fraction analysis method (MSFA, Wei et al., 2019) has been developed. We found that it can improve the classification accuracy in some circumstances, but that it does not provide an advantage for the system we present in the manuscript. Whereas the application of data processing methods which have

Vorstandsmitglieder:
Prof. Dr. Burkhard Göke
(Vorstandsvorsitzender)
Prof. Dr. Dr. Uwe Koch-Gromus
Joachim Prölß
Rainer Schoppik

Gerichtsstand: Hamburg
Körperschaft des öffentlichen Rechts
USt-Id: DE 21 8618 948

Bank: HSH Nordbank
BLZ: 210 500 00 | Konto: 104 364 000
IBAN: DE9721 0500 0001 0436 4000
BIC: HSHNDEHH

Universitätsklinikum Hamburg-Eppendorf

shown to be useful in motor-imagery BCIs for SSVEP BCI is certainly an interesting idea, we suggest exploring their potential in a future study.

4. *For BCI system, signal decomposition methods always play significant role. I recommend authors to have a look on following article "Motor Imagery EEG Signals Classification Based on Mode Amplitude and Frequency Components Using Empirical Wavelet Transform"*

See 5.

5. *Did authors try to use non-linear features for correct identification in BCI? I recommend authors to include discussion of mean energy, mean Teager-Kaiser energy, SHANNON WAVELET ENTROPY and Log energy entropy.*

The correlation features we used in the study yielded excellent classification results in our previous studies on this paradigm, and similar features are frequently used in other SSVEP BCI systems. We are always interested in evaluating alternative methods which can increase the classification accuracy. The focus of our current manuscript however is on dynamic stopping; therefore, we suggest comparing different methods for feature extraction in a separate study.

6. *The combination of signal decomposition with dimension reduction techniques along with neural networks can be one effective tool for both subject dependent and independent BCI frameworks. Authors need to discuss this issue; detail may be found in "Exploiting dimensionality reduction and neural network techniques for the development of expert brain–computer interfaces".*

The recommended article proposes LDA as one of several methods for dimension reduction, and we employ this method in our approach for classification. We would like to point out that the features calculated by eq. (1) are 10-dimensional. Compared to the problem which is analyzed in the recommended article, this already is a rather low-dimensional feature space. Whereas the development of a user-independent version of the spatially-coded SSVEP BCI is an interesting endeavor, it was not the focus of the current study.

7. *The authors recorded dataset from very few subjects. Is it possible to collect dataset from more subjects? If it is not possible, at least a discussion is needed for a framework tested on 58 subjects. See following article "Towards the development of versatile brain-computer interfaces"*

A main difference between the recommended article and our study is that we ran an online experiment with participants in our laboratory. Hence, we would like to compare the number of participants in our study with related studies on dynamic stopping which also included an online experiment:

Reference in the manuscript	Number of participants
3	11
5	12

6	14
7	12
8	12
24	10
25	10
**Our study**	**14**

In the light of these numbers, it seems that the size of our cohort is quite standard.

8. *Please provide a comprehensive comparison of your study with the available literature in terms of classification accuracy, number of channels, features, and execution time with the following articles,*

"A new framework for automatic detection of motor and mental imagery EEG signals for robust BCI systems", "A Matrix Determinant Feature Extraction Approach for Decoding Motor and Mental Imagery EEG in Subject Specific Tasks", "Motor imagery BCI classification based on novel two-dimensional modelling in empirical wavelet transform", "Identification of Motor and Mental Imagery EEG in Two and Multiclass Subject-Dependent Tasks Using Successive Decomposition Index"

All the recommended articles seem to study MI BCIs, which involve different task instructions for the operator and employ other signal analysis methods than SSVEP BCIs. We think, therefore, that a comparison of our paradigm with (frequency-coded) SSVEP BCIs and in particular BCIs with a dynamic trial duration would be more appropriate. We added a respective table to the Discussion.

9. *Please provide the details of future direction and possible solutions to continue this topic.*

We mention a number of suggestions for future developments and studies at the end of the Discussion.

10. *Finally, I suggest authors to sit with English native speaker to improve the writing of proposed work.*

We overhauled the manuscript, trying to improve style and fix grammar errors. Should concerns about our writing persist, a few examples for which aspects need improvement would be appreciated.

We hope that these modifications make the manuscript acceptable for publication.

Best regards,

A. Maÿe

Alexander Maÿe.
