# Supplementary material for: Instant classification for the spatially-coded BCI
Source: PLoS One. 2022 Apr 28;17(4):e0267548. doi: 10.1371/journal.pone.0267548 (PMC9049359; doi:10.1371/journal.pone.0267548)
Supplement: Attachment — Submitted filename: response to reviewers 3.pdf [file pone.0267548.s002.pdf]

Zentrum für Experimentelle Medizin
Institut für Neurophysiologie und Pathophysiologie

Dr. Alexander Maÿe
Wissenschaftlicher Mitarbeiter

Martinistraße 52
20246 Hamburg

Universitätsklinikum Hamburg-Eppendorf | Martinistraße 52 | 20246 Hamburg
Institut für Neurophysiologie und Pathophysiologie, N43

Gebäude Nord 43, MEG Büro 2
Raum 005
Telefon: +49 (0) 40 7410-55352
Fax: +49 (0) 40 7410-57752
a.maye@uke.de
www.uke.de

PloS ONE
Editor in Chief

Betreff: Revised manuscript PONE-D-21-13493

Hamburg, 7.4.2022

Dear Editor,

Thank you very much for reviewing our manuscript again. The new comments may help improving the manuscript indeed, and we changed it as described below (reviewer comments in italics):

1. Can you describe the workflow of the stimulus interface in detail?

The workflow in the training session is described on lines 100–108 and in the online session on lines 114--129 in the section „Stimulation and experimental procedure“. The interface only generated the visual flicker (line 91) and a cue (line 101, Figure 1) which directed the participant’s gaze to the respective target location (numbered 1-5, see Fig. 1) relative to the flicker. We cannot think of any other details about the stimulation or the workflow that might be missing, but we will readily add them if the reviewer makes a more specific request.

2. We noticed that the experimental results between the best subjects and the worst subjects are very different. What is the reason for this?

There are several studies which investigated possible causes for the strong variability in BCI performance across people (e.g., Allison et al., BCI Demographics: How Many (and What Kinds of People) Can Use an SSVEP BCI?, IEEE TNSRE 18(2), 2010), but as far as we know, no factors have been identified. We made an attempt to contribute to this line of research and show in Fig. 7 that the ITR is related to the individual SSVEP response strength of the participants. Of course the next question then is what the reason for this difference in response strength might be. We regret to not have an answer. We nevertheless tried to better understand the observed performance difference and rearranged the bar plot in Fig. 5 by sorting the participants according to their ITR in the online session. The new arrangement suggests that the two subjects with the lowest performance are part of a continuum rather than outliers.

3. Please list the promotion ratio in Table 2 to improve the readability of the paper.

We added the corresponding values to Table 2.

4. The serial number is not indicated in Figure 5 to Figure 7 in the picture area.

This was a problem during the submission process which we solved now, so no changes were necessary in the manuscript.

Vorstandsmitglieder:
Prof. Dr. Burkhard Göke
(Vorstandsvorsitzender)
Prof. Dr. Dr. Uwe Koch-Gromus
Joachim Prölß
Rainer Schoppik

Gerichtsstand: Hamburg
Körperschaft des öffentlichen Rechts
USt-Id: DE 21 8618 948

Bank: HSH Nordbank
BLZ: 210 500 00 | Konto: 104 364 000
IBAN: DE9721 0500 0001 0436 4000
BIC: HSHNDEHH

Universitätsklinikum Hamburg-Eppendorf

5. Is there a significant difference between the test results of the offline test and the online test after using the dynamic time window?

We added a comparison of classification accuracies and ITRs to the Results section.

6. A study needs to be discussed in this study, such as "A Dynamically Optimized SSVEP Brain–Computer Interface (BCI) Speller[J]. IEEE Transactions on Biomedical Engineering, 2015, 62(6): 1447-1456.".

The manuscript discusses this article on line 27–29, lists the results in Table 2 and cites it in the references [3]. Nevertheless, we added a few more details on lines 30--32.

We hope that these modifications address the concerns properly make the manuscript acceptable for publication.

Best regards,

Alexander Maÿe.
